# Citronellal Exerts Sedative‐*Like* Effects and Augments Diazepam's Action in *Swiss* Mice, Possibly Through the GABAergic Pathway

**DOI:** 10.1002/brb3.70446

**Published:** 2025-03-23

**Authors:** Md. Torequl Islam, Md. Sakib Al Hasan, Emon Mia, Irfan Aamer Ansari, Siddique Akber Ansari, Md. Amirul Islam, Md. Saifuzzaman

**Affiliations:** ^1^ Pharmacy Discipline Khulna University Khulna Bangladesh; ^2^ Department of Pharmacy Gopalganj Science and Technology University Gopalganj Bangladesh; ^3^ Bioinformatics and Drug Innovation Laboratory BioLuster Research Center Ltd. Gopalganj Bangladesh; ^4^ Department of Drug Science and Technology University of Turin Turin Italy; ^5^ Department of Pharmaceutical Chemistry, College of Pharmacy King Saud University Riyadh Saudi Arabia; ^6^ Department of Pharmacy East West University Dhaka Bangladesh

**Keywords:** citronellal, GABAkine pathway, monoterpene aldehyde, sedative effect

## Abstract

**Introduction:**

Citronellal (CTL), a monoterpenoid, exhibits notable neurological activity, including anxiolytic, and anticonvulsant effects, primarily through GABAergic pathways. Our current study aimed to explore CTL's sedative potential using in vitro, in vivo, and in silico approaches through the GABAergic pathway.

**Methods:**

The in vitro GABAergic activity of CTL was assessed via colorimetric assay, while acute toxicity was evaluated in *Swiss* mice per OECD guidelines with doses up to 2000 mg/kg to establish safety margins. Sedative effects were assessed in *Swiss* mice using thiopental sodium (TS, 40 mg/kg)‐induced sleep protocols. CTL was administered at 62.5, 125, and 250 mg/kg doses, alone or combined with diazepam (DZP, 2 mg/kg) or flumazenil (FLU, 0.1 mg/kg). The in silico studies were also performed with GABA_A_ receptors (α1 and β2 subunits) to investigate the possible molecular mechanism.

**Results:**

The results demonstrated that in vitro, CTL exhibited significantly concentration‐dependent GABAergic activity. Acute toxicity tests indicated a high safety margin (no behavioral or physiological abnormalities at 2000 mg/kg dose). Additionally, CTL significantly (*p* < 0.05) and dose‐dependently reduced the latency and augmented sleep duration in animals, compared to the control group. It also significantly (*p* < 0.05) decreased the latency and increased the duration of sleep with DZP‐2 while reducing this parameter with FLU‐0.1. In in silico studies, CTL exhibited binding affinities (BAs) with the GABA_A_ receptor (α1 and β2 subunits) of –5.6 kcal/mol.

**Conclusion:**

CTL demonstrated potent sedative effects in vitro and in vivo, with a strong safety profile and interaction with the GABA_A_ receptor (α1 and β2 subunits).

## Graphical abstract

Citronellal (CTL) alone dose‐dependently or with the GABA agonist drug diazepam significantly (*p* < 0.05) reduced the latency and augmented sleep duration, while altering these parameters with flumazenil in thiopental sodium‐induced sleeping mice, and it showed a binding affinity with 6X3X of the GABA_A_ receptor of –4.7 kcal/mol.

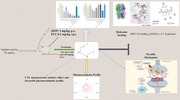

**Abbreviations**
ADMET: absorption, distribution, metabolism, excretion, and toxicity
ANOVA: analysis of variance
BA: binding affinity
CAN: central nervous system
CTL: citronellal
DZP: diazepam
FLU: flumazenil
GABA: gamma‐aminobutyric acid
LD50: median lethal dose
SCN: suprachiasmatic nucleus
SD: standard deviation

## 1 Introduction

Insomnia is a neurological condition that is a type of sleep disorder normally treated with sedative agents (Benington et al. 1995). Sleep disorders, including insomnia, are one of the major global burdens that require continuous new remedies. It is because these kinds of medical conditions cause some common adverse effects, such as tolerance and dependence on the treatments for long‐term use. At present, there is a lack of suitable drugs for the management of chronic insomnia (Olayiwola et al. 2013). In this case, patients develop tolerance rapidly to the existing sedatives, thereby leading them to select high doses of another potent drug or a mixture of medications. The ultimate result is mild to serious side effects and a worsening insomnia condition in this class of patients (Morrissettee and Heller 1998).

Gamma‐aminobutyric acid (GABA), a major inhibitory neurotransmitter in our brain, is involved in various neuronal signaling pathways. An increased GABAergic transmission is evident to produce profound sedation in experimental animals (e.g., mice, rats; Gottesmann 2002). It is evident that the sedative/hypnotic effects are mediated by the 6 × 3X protein of the α1 and β2 subunits of the GABA_A_ receptor (Reynolds et al. 2003; Nilsson and Sterner 2011). Thus, targeting these subunits of the GABA_A_ receptor might be an interesting approach for the development of new and effective sedative molecules of various origins, including natural products and their derivatives.

Many drugs acting through the central nervous system (CNS), especially through GABA receptors, can exert both anxiolytic and sedative effects on animals, for example, benzodiazepines (BDZs; Khan et al. 2014). A number of studies demonstrate that the essential oils of *Citrus limon* (Lopes et al. 2011) and *Citronella* (Yulianita et al. 2019; Vitamia and Handayani 2024) exert sedative effects on experimental animals. Citronellal (CTL) is a monoterpenoid aldehyde, which is the main component of *Citronella* oil and possesses many health benefits, including neurological activities. An earlier report also suggests that CTL reduced movement and thereby exerted a sedative‐like effect on *Swiss* mice (Jäger et al. 1992). Drugs having sedative and analgesic properties are used in the care of critically ill, mechanically ventilated patients. Sedatives with amnestic properties are desirable to prevent or relieve anxiety and agitation. In this case, BDZs and propofol are the primary sedative agents used in the intensive care unit (Wong et al. 2004). Several studies report that CTL has anti‐nociceptive effects on experimental animals (Melo et al. 2010; Costa et al. 2023). Another study showed that CTL exhibited CNS effects in rodents, including anxiolytic and anticonvulsant properties, as revealed through bioassay‐guided evaluation (Melo et al. 2011). In addition, CTL demonstrated anticonvulsant effects potentially through GABAergic and voltage‐gated sodium channel receptor interactions (Chowdhury et al. 2024). A recent study revealed that CTL exhibited significant anxiolytic and analgesic effects in *Swiss* mice, mediated through COX and GABA_A_ receptor pathways (Islam et al. 2024). Thus, it is a time‐ask to check the sedative potentials of CTL.

Knowing the overall facts, the current study aimed at evaluating the sedative effect of CTL using a thiopental sodium (TS)‐induced sleeping protocol in mice. To understand the possible molecular mechanism behind this neurological effect of CTL, we combined it with a GABAergic agonist (DZP) and an antagonist (FLU) drug and performed in silico studies with GABA_A_ receptor‐responsible subunits.

## 2 Materials and Methods

### 2.1 Colorimetric GABAergic Activity Analysis

This study was done according to an earlier described model by Jinnarak and Teerasong (2016) with some modifications. For this, a stock solution of 2 g/L of GABA (from Gabarol50) was prepared by dissolving 200 mg of GABA in 100 mL of distilled water. The solution was then filtered and kept in a conical flask. A volume of 1 mL of acetate buffer was added to 1 mL of GABA solution and kept in individual test tubes containing 1 mL of test sample CTL with different concentrations (15.625, 31.25, 62.50, 125, and 250 µg/mL). As positive controls, DZP (GABA agonist: 0.125, 0.25, 0.5, 1, and 2 µg/mL) and FLU (GABA antagonist: 0.00625, 0.0125, 0.025, 0.05, and 0.1 µg/mL) were used at a specified series of concentrations. The final volume of 5 mL mark was adjusted with DW. DW was considered a negative control for this assay. To prepare 0.5 M acetate buffer at a pH of 3.8, 50 mL of 0.5 M (3.402 g) sodium acetate trihydrate was mixed with 450 mL of 0.5 M acetic acid (30.026 g). The pH of the solution was adjusted to 3.8 by the addition of NaOH or HCl. Then the reaction mixture was vortexed for 20 s and observed for color change. Finally, the optical density of the mixture was measured at 620 nm using a colorimeter (LT‐114, India). The percentage GABA content was determined by using the following formula:

$$\begin{array}{ccc} & & \mathrm{Percentage}\mathrm{of}\mathrm{GABA}\mathrm{activity}=100\\ & & \;-\;\left[\left\{\left(\mathrm{OD}\mathrm{of}\mathrm{Control}-\mathrm{OD}\mathrm{of}\mathrm{Test}\right)\;÷\mathrm{OD}\mathrm{of}\mathrm{Control}\right\}\times 100\right] \end{array}$$

The half‐minimal inhibitory concentration (IC_50_) values for the test sample and/or standards were also determined using non‐linear regression analysis in GraphPad Prism.

### 2.2 In Vivo Approach

#### 2.2.1 Chemicals and Reagents

(±) CTL (CAS: 106‐23‐0, purity: > 95%, GC) was bought from Sigma‐Aldrich, Germany, while the standard drug DZP and the inducer TS were kindly provided from Square Pharmaceuticals Ltd., Bangladesh, while flumazenil (FLU) was purchased from Centurion Healthcare Private Ltd., India, respectively. Tween 80 and NaCl required for this study were purchased from Merck (India).

#### 2.2.2 Experimental Animals

Adult male *Mus musculus* (*Swiss* mice; avg. b.w. 24–30 g) purchased from the Animal House of Jahangirnagar University, Bangladesh, were randomly divided into different groups of six animals each. Before that, the animals were housed in standard conditions (temperature: 26°C ± 2°C, relative humidity: 65%) for 7 days. They had free access to standard foods and water ad libitum. Experiments were conducted during the same period of the light–dark cycle to account for potential circadian influences, circadian rhythms, and environmental factors on sleep behavior. This study was approved by the Animal Ethics Committee of Khulna University (KUAEC‐2023‐05‐09).

#### 2.2.3 Acute Toxicity Study, Selection, and Administration of Test Doses

According to the PubChem database, CTL showed an LD_50_ of 2420 mg/kg (p.o.) in rats and > 200 mg/kg (i.p.) in mice (https://pubchem.ncbi.nlm.nih.gov/compound/Citronellal#section = Acute‐Effects). The present investigation used *Swiss* mice to conduct an acute toxicity study for CTL according to OECD guidance. The CTL was given to the patients orally (p.o.) at 500, 1000, and 2000 mg/kg. The animals were subsequently monitored for 2 days in order to look for behavioral changes, toxicological symptoms, and mortality (Kamli et al. 2023). The test doses for CTL (62.5, 125, and 250 mg/kg) in mice were selected according to previous literature studies (Melo et al. 2011, 2010; Islam et al. 2024). DZP was administered at 2 mg/kg, while the control (vehicle: distilled water containing 0.9% NaCl and 0.5% tween 80). All treatments were administered at a volume of 10 mL/kg. Before treatment, all animals were fasted overnight. The mice were randomly categorized into different groups, each comprising six animals. FLU and DZP were administered via the intraperitoneal route (i.p.), while CTL was given via oral gavage (p.o.) before 30 min of starting the study. TS was administered at 40 mg/kg via the i.p. route 30 min after each treatment. Then, follow the below‐mentioned study.

#### 2.2.4 Sedative Effect Study in *Swiss* Mice

##### 2.2.4.1 Study Design

In this study, a total of 54 adult male *Swiss* mice were randomly divided into nine groups, each containing six animals (*n* = 6). Treatment groups and their details have been shown in Table 1.

**TABLE 1:** Treatment groups with their details at 10 mL/kg volume of administration.

Treatment groups	Description	Administration design
**Individual groups**		
Control	Vehicle: Distilled water containing 0.9% NaCl and 0.5% tween 80	p.o., at a time
DZP‐2	Diazepam (GABA agonist reference drug) at 2 mg/kg	i.p., at a time
FLU‐0.1	Flumazenil (GABA antagonist reference drug) at 0.1 mg/kg	i.p., at a time
CTL‐62.5	Citronellal (test sample) at 62.5 mg/kg	p.o., at a time
CTL‐125	Citronellal (test sample) at 125 mg/kg	p.o., at a time
CTL‐250	Citronellal (test sample) at 250 mg/kg	p.o., at a time
**Combination groups**		
CTL‐250+DZP‐2	Citronellal 250 mg/kg + diazepam 2 mg/kg	p.o.+i.p., one followed by another
CTL‐250+FLU‐0.1	Citronellal 250 mg/kg + flumazenil 0.1 mg/kg	p.o.+i.p., one followed by another
CTL‐250+DZP‐2+FLU‐0.1	Citronellal 250 mg/kg + diazepam 2 mg/kg + flumazenil 0.1 mg/kg	p.o.+i.p.+i.p., one followed by another

*Note*: Control: vehicle (distilled water containing 0.9% NaCl and 0.5% tween 80).

Abbreviations: CTL: citronellal; DZP: diazepam; FLU: flumazenil; i.p.: intraperitoneal; p.o.: per oral (*n* = 6).

After TS treatment, each animal was observed for the loss of righting reflex (onset of sleep, i.e., latency in min) and the time of total recovery of righting reflex (sleep duration in min) up to 4 h.

#### 2.2.5 TS‐Induced Sleeping Study in Mice

This study was carried out according to the method described by Turner (2013), with slight modifications (Turner 2013). The test sample (CTL) and control (vehicle: 10 mL/kg) were administered orally (p.o.), while the GABA agonist DZP (2 mg/kg) and antagonist FLU (0.1 mg/kg) were given via the intraperitoneal (i.p.) route. After 30 min, sleep inducer TS (40 mg/kg, i.p.) was administered to each mouse and observed for the latency (time between TS administration and loss of righting reflex) and sleep duration (time between the loss and recovery of reflex).

#### 2.2.6 Statistical Analysis

Values are expressed as the mean ± SD (standard deviation). One‐way analysis of variance (ANOVA) followed by *t*‐students Newman–Keuls as a post hoc test with multiple comparisons at 95% confidence intervals using GraphPad Prism software (version: 9.5, San Diego). Data were considered significant when *p <* 0.05. The Shapiro–Wilk test confirmed that the data followed a normal distribution (*p* > 0.05), and Levene's test indicated no significant variance heterogeneity across groups (*p* > 0.05). These checks validated the use of one‐way ANOVA and post hoc Newman–Keuls multiple comparison tests.

### 2.3 In Silico Approach

#### 2.3.1 GABA Macromolecule Selection

The Protein Data Bank (PDB) is a global library that offers free, open access to experimentally established 3D structures of molecules in biology (Burley et al. 2022). The three‐dimensional structures of GABA_A_ (PDB ID: 6 × 3X; α1 and β2) receptor subunits (Bhuia et al. 2024), crucial for understanding their function, were acquired from the RCSB PDB (Kim et al. 2020).

#### 2.3.2 Protein Preparation

To optimize the receptors for docking, irrelevant components were removed from the macromolecular structures (Vakser 1996). Specifically, PyMOL version 1.7.4.5 was employed to eliminate extraneous amino acid residues, heteroatoms, and water molecules (Chowdhury et al. 2023). Protein structure energy was minimized using the GROMOS96 43 B1 force field within the Swiss‐PDB Viewer software (version 4.1.0; Solayman et al. 2017).

#### 2.3.3 Ligand Preparation

To prepare for the docking simulations, 3D structures of the ligands were retrieved from the PubChem online database (https://pubchem.ncbi.nlm.nih.gov/). This included CTL (PubChem ID: 7794) and two standard drugs, FLU (PubChem ID: 3373) and DZP (PubChem ID: 3016). The structures were downloaded in SDF format. Using the Chem3D Pro 20.1.1 software, we optimized the ligands through energy minimization using the MM2 (Allinger's force field) technique (Bappi et al. 2023; Al Hasan et al. 2024b).

#### 2.3.4 Docking Protocol and Non‐Bond Interactions

Molecular docking is a common computational method in pharmaceutical studies to evaluate and align molecules to specific binding sites, assessing their pharmacodynamic properties using software like PyRx v0.8 (Prottay et al. 2024). This approach helps in determining how well a ligand binds to the active site of a particular protein. The strength of this binding provides insights into the potential efficacy of the medication being studied. To speed up the docking process, the grid box dimensions along the X, Y, and Z axes were set to their maximum values (Chowdhury et al. 2024). The calculation was subsequently performed in 200 steps. The PDB format of the ligand–protein complex was obtained to extract the ligand in PDBQT format. (Agrawal et al. 2024). An experiment analyzing the active binding regions of proteins is conducted using BIOVIA Discovery Studio v21.1.0, which aids in examining non‐bond interactions within ligand–protein complexes. (Bhuia et al. 2023; Al Hasan et al. 2024a).

#### 2.3.5 Pharmacokinetics and Drug‐Likeness Properties

PkCSM is an online computational tool used in this study to assess pharmacokinetic (PK) properties and predict potential toxicity (Pereira et al. 2024). It assesses a medication's absorption, distribution, metabolism, excretion, and toxicity (ADMET) in the body, which aids in the optimization of drug candidates before clinical testing (Sucharitha et al. 2022). Additionally, the SwissADME web tool was used to examine the drug‐likeness (Anebi et al. 2021).

#### 2.3.6 Toxicity Profiling

ProTox‐3.0 (https://tox.charite.de/protox3/) is an online tool that predicts the toxicity of chemical compounds, including acute toxicity, hepatotoxicity, carcinogenicity, and other adverse effects (Banerjee et al. 2025; Islam et al. 2025). For determining the toxicity of selected ligands, this ProTox‐3.0 (Noga et al. 2024) server was used.

## 3 Results

### 3.1 In Vitro Findings

The control group (vehicle) exhibited negligible GABAergic activity (4.75% ± 0.01%). In comparison, CTL and DZP concentration‐dependently and significantly (*p* < 0.05) enhanced GABAergic activity in test tubes. At high concentrations, CTL (250 µg/mL) and DZP (2 µg/mL) exhibited GABAergic activities of 71.43% ± 0.01% and 72.50% ± 0.01%, respectively. In contrast, FLU demonstrated a unique pattern, concentration‐dependently but significantly (*p* < 0.05), reducing GABAergic activity at higher concentrations. However, at the lowest concentration (0.00625 µg/mL), FLU exhibited the highest GABAergic activity (55.00 ± 0.02%). The IC_50_ values calculated for CTL, DZP, and FLU were 120.86 ± 1.29 µg/mL, 0.87 ± 0.43 µg/mL, and 0.005 ± 0.001 µg/mL, respectively, with confidence intervals and R^2^ values indicating a reliable fit (Table 2).

**TABLE 2:** In vitro GABAergic activity of test sample and the standard drugs.

Treatment with concentration		Percentage GABAergic activity	IC_50_ [CI; R^2^]
Control (vehicle)	1 mL	4.75 ± 0.01	—
CTL	15.625 µg/mL	28.57 ± 0.01^*^	120.86 ± 1.29 µg/mL (108.43–140.03 µg/mL; 0.98)
	31.25 µg/mL	35.71 ± 0.01^*^	
	62.5 µg/mL	42.86 ± 0.01^*^	
	125 µg/mL	57.14 ± 0.01^*^	
	250 µg/mL	71.43 ± 0.01^*^	
DZP	0.125 µg/mL	17.50 ± 0.01^*^	0.87 ± 0.43 µg/mL (0.84–1.59 µg/mL; 0.95)
	0.25 µg/mL	27.50 ± 0.01^*^	
	0.5 µg/mL	37.50 ± 0.01^*^	
	1 µg/mL	57.50 ± 0.01^*^	
	2 µg/mL	72.50 ± 0.01^*^	
FLU	0.00625 µg/mL	55.00 ± 0.02^*^	0.005 ± 0.001 µg/mL (0.003–0.006 µg/mL; 0.94)
	0.0125 µg/mL	37.50 ± 0.02^*^	
	0.025 µg/mL	32.50 ± 0.02^*^	
	0.05 µg/mL	20.00 ± 0.01^*^	
	0.1 µg/mL	12.50 ± 0.01^*^	

*Note*: Values are mean percentage ± SD (*n* = 3); one‐way ANOVA followed by Tukey post hoc test with single comparison.

Abbreviations: CTL: citronellal; DZP: diazepam; FLU: flumazenil; IC_50_: 50% inhibitory concentration; CI: confidence of intervals; R^2^: co‐efficient of determination.

* *p <* 0.05, compared to the control (vehicle: distilled water containing 0.9% NaCl and 0.5% tween 80).

### 3.2 In Vivo Findings

#### 3.2.1 Acute Toxicity Findings

In preclinical research, acute toxicity testing is crucial for choosing safe test concentrations and doses for experimental animals (Kamli et al. 2023). In the acute toxicity evaluation, mice were given oral dosages of CTL up to 2000 mg/kg without experiencing any discernible behavioral abnormalities, toxicity, or mortality. Therefore, in *Swiss* mice, the LD_50_ of CTL may be higher than 2 g/kg oral dosage. Our experimental results and literature studies led us to choose the highest test dose of 250 mg/kg for CTL, which is then halved at 62.5 mg/kg as the lowest test dose.

#### 3.2.2 TS‐Induced Sleeping Time Test

**FIGURE 1:**
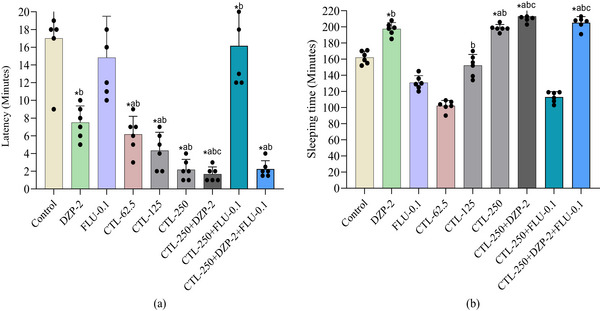
(a) Latency and (b) sleeping time in minutes observed in different treatment groups of animals. (values are mean ± SD [*n* = 6]; one‐way ANOVA followed by *t*‐students Newman–Keuls as a post hoc test with multiple comparisons; *p <* 0.05, compared to the *control, ^a^DZP‐2, ^b^FLU‐0.1, and ^c^CTL‐250 group; control: vehicle (distilled water containing 0.9% NaCl and 0.5% tween 80); CTL: citronellal; DZP: diazepam; FLU: flumazenil).

In this study, we have seen that all groups produced an onset of sleep within 1.67–14.83 min and showed a maximum sleep of 3.55 h. No animals had gone into a coma, and no animals died during the observation period (4 h) or even after 48 h of the experiment. The control group exhibited a latency of 17.00 ± 4.15 min, while the standard agonist drug DZP (2 mg/kg) exhibited a latency of 97.50 ± 1.87 min, and the antagonist drug FLU (0.1 mg/kg) exhibited a latency of 14.83 ± 4.67 min in TS‐induced sleeping animals. CTL at all doses showed better latency reduction capacity than all control groups. It dose‐dependently and significantly (*p* < 0.05) reduced the latency in animals. CTL‐250 combined with DZP‐2 remarkably and significantly (*p* < 0.05) decreased the latency value (1.67 ± 0.82 min), which was the lowest value, compared to all treatment groups. However, PHY‐75, when treated with FLU‐0.1, augmented the latency value (16.17 ± 4.40 min) in animals, which was subsequently reduced to 2.25 ± 0.94 min by the addition of DZP‐2 with this combined group (CTL‐250+DZP‐2+FLU‐0.1; Figure 1a).

In the sleep duration parameter measurement, the control group exhibited a sleep duration of 162.00 ± 7.90 min. The standard agonist group DZP‐2 significantly (*p* < 0.05) increased latency (197.50 ± 7.87 min), while the antagonist drug FLU (0.1 mg/kg) significantly (*p* < 0.05) reduced (130.83 ± 8.75 min) this parameter when compared to the control group. In this case, CTL also showed a dose‐dependent sleep duration in animals. However, CTL at 250 mg/kg only exhibited a significant (*p* < 0.05) sleep duration in comparison to the control and DZP‐2 groups. CTL at 250 mg/kg showed a sleep duration of 198.50 ± 4.46 min. However, CTL‐250 co‐treated with DZP‐2 profoundly and significantly (*p* < 0.05) augmented the sleep duration in animals (213.17 ± 9.43 min), which was the highest sleep duration among all treatment groups. The combination group CTL‐250+DZP‐2+FLU‐0.1 also produced a significant (*p* < 0.05) sleep duration in comparison to all groups except the combination group CTL‐250+DZP‐2 (Figure 1b).

### 3.3 In Silico Findings

#### 3.3.1 Molecular Docking and Visualization

In our in silico study, CTL exhibited a BA of ‒5.6 kcal/mol with interactions involving PHE A: 289, LEU A: 285, PRO B: 233, MET B: 236, MET A: 261, and LEU B: 232 but no hydrogen bonds (HBs). DZP showed the strongest BA at ‒8.7 kcal/mol, forming one HB with LEU A: 285 and interacting with residues PHE A: 289, PRO A: 233, MET A: 236, MET A: 286, LEU A: 285, and LEU A: 232. FLU demonstrated a BA of ‒6.9 kcal/mol, forming seven HBs with ARG A: 18, ASP B: 10, ASN B: 11, THR B: 12, LEU A: 19, GLY A: 22, and LYS A: 70, alongside additional interactions with ARG A:18, LYS A:70, and ARG A:71.

**FIGURE 2:**
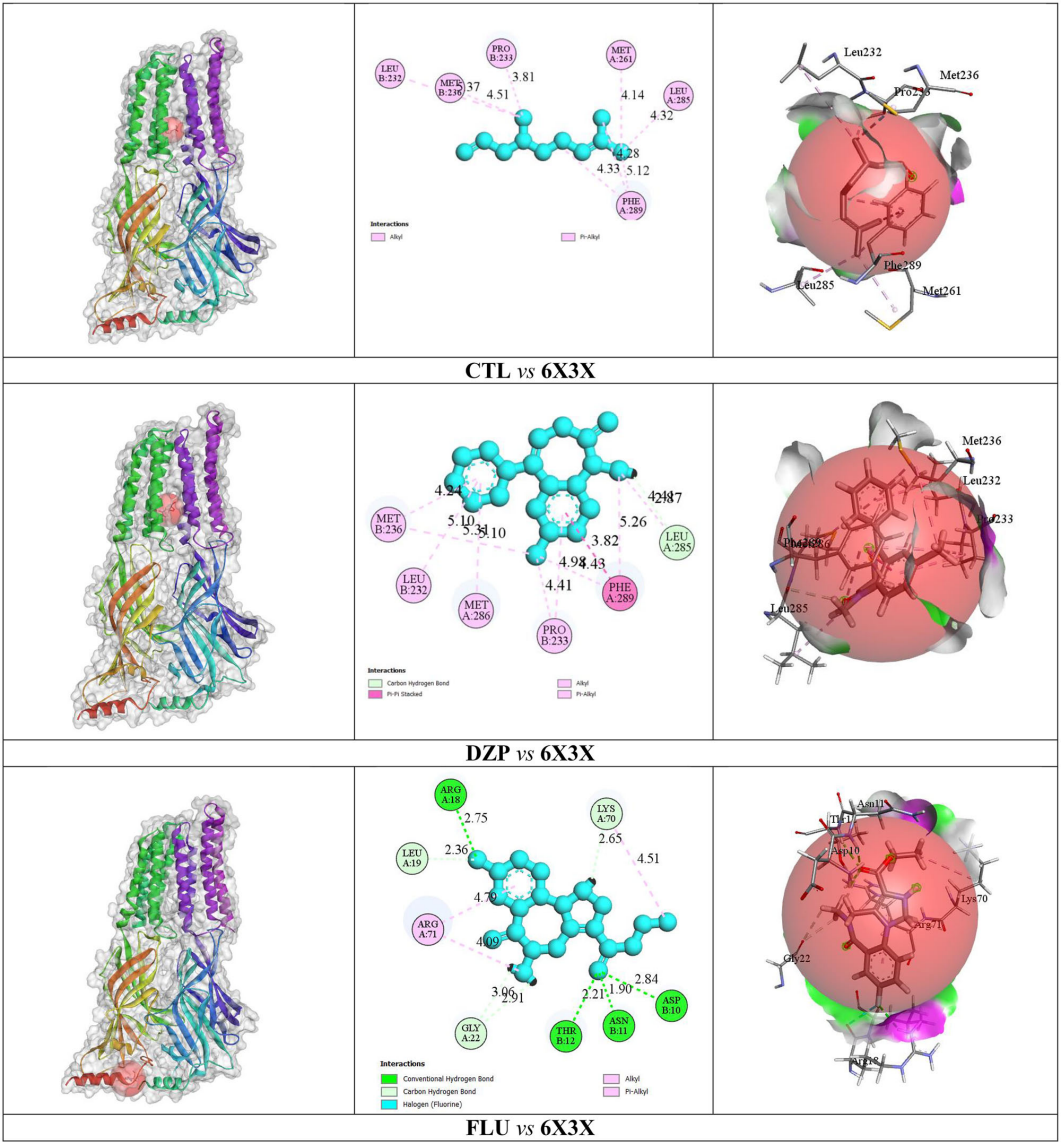
The 2D and 3D non‐bond interaction between citronellal, diazepam, and flumazenil with the GABA_A_ receptor (α1 and β2 subunits).

However, details of the BA, number of HB and AA residues related to HB, and other types of bonds of ligands with the GABA_A_ receptors (α1 and β2 subunits) are presented in Table 3. The binding pockets in 2D and 3D structures, including the interacting AA residues and bond types of CTL, DZP, and FLU with the GABA_A_ receptors (α1 and β2 subunits), are depicted in Figure 2.

**TABLE 3:** Binding affinity and the amino acid interactions between citronellal, diazepam, and flumazenil with GABA_A_ receptors (α1 and β2 subunits).

Ligands	Receptors (PDB ID)	BA (kcal/mol)	No of HB	Amino acid (AA) residues	
				HB	Others
CTL	GABA_A_ receptors (α1 and β2 subunits) (6 × 3X)	‒5.6	—	—	PHE A: 289, LEU A: 285, PRO B: 233, MET B: 236, MET A: 261, LEU B: 232
DZP		−8.7	1	LEU A: 285	PHE A: 289, PRO A: 233, MET A: 236, LEU A: 285, MET A: 286, LEU A: 232
FLU		‒6.9	7	ARG A: 18, ASP B: 10, ASN B: 11, THR B: 12, LEU A: 19, GLY A: 22, LYS A: 70	ARG A: 18, LYS A: 70, ARG A:71

Abbreviations: BA: binding affinity; CTL: citronellal; DZP: diazepam; FLU: flumazenil; HB: hydrogen bonds.

#### 3.3.2 Pharmacokinetics and Drug‐Likeness Properties

In our study, the molecular weights of the molecules CTL, DZP, and FLU, which are all under 500, were found to be 154.25, 284.74, and 303.29 g/mol, respectively, according to the prediction. CTL and FLU are water‐soluble, and DZP is moderately soluble. On the other hand, all compounds have high GI absorption and are BBB permeable. DZP is the only substance that inhibits CYP2C19. The drug‐like qualities demonstrate that all compounds follow the Lipinski rule. All compounds containing TPSA are also within limits (TPSA ≤ 140 Å).

**FIGURE 3:**
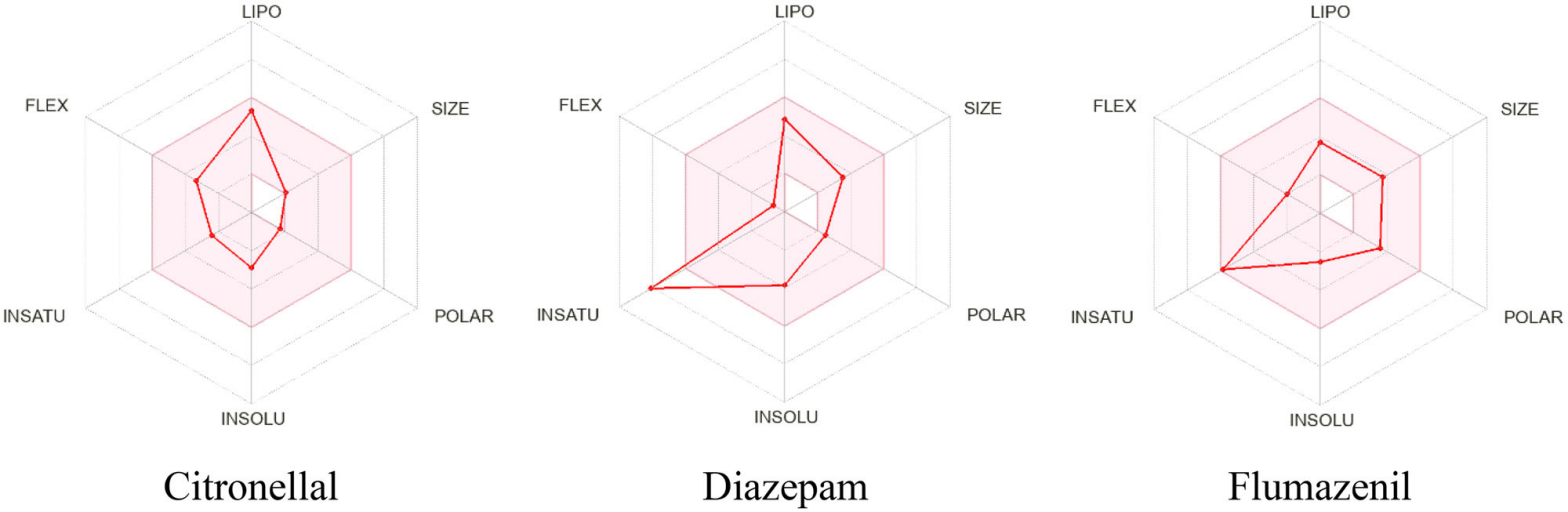
Summarizes the selected chemical's physiochemical characteristics. (The physicochemical space that is appropriate for oral bioavailability is the colored zone; SIZE: 150 g/mol < MV < 500 g/mol; LIPO [lipophilicity]: –7 < XLOGP3 < + 5.0; INSOLU [insolubility]: –6 < log S [ESOL] < 0; POLAR [polarity]: 20 Å^2^ < TPSA < 130 Å^2^; IN‐SATU [in saturation]: 0.25 < Fraction Csp3 < 1; FLEX [flexibility]: 0 < num. rotatable bonds < 9.)

In addition, Table 4 lists additional factors, including P‐gp substrate, synthetic accessibility, and bioavailability score. Figure 3 shows a graphical representation of the physiochemical and PK properties of particular compounds.

**TABLE 4:** Pharmacokinetics properties of citronellal, diazepam, and flumazenil.

Parameters	CTL	DZP	FLU
**Physicochemical properties**			
MF	C_10_H_18_O	C_16_H_13_ClN_2_O	C_15_H_14_FN_3_O_3_
MW	154.25 g/mol	284.74	303.29 g/mol
No of heavy atoms	11	20	22
No of aromatic heavy atom	0	12	11
HBA	1	2	3
HBD	0	0	5
TPSA (Å^2^)	17.07 Å^2^	32.67 Å^2^	64.43 Å^2^
MR	49.91	87.95	79.47
**Solubility**			
Solubility (water)	Soluble	Moderately soluble	Soluble
**Lipophilicity**			
Log P*_o/w_* (XLOGP3)	3.83	2.99	1.00
Log P*_o/w_* (WLOGP)	2.96	2.39	1.66
**Pharmacokinetics**			
GI absorption	High	High	High
BBB permeant	Yes	Yes	Yes
P‐gp substrate	No	No	No
CYP2C19 int.	No	Yes	No
**Medicinal chemistry**			
Synthetic accessibility	2.57	3.00	3.06
**Drug‐*likeness***			
Lipinski	Yes; 0 violation	Yes; 0 violation	Yes; 0 violation
BIO score	0.55	0.55	0.55

Abbreviations: BIO score: bioavailability score, CTL: citronellal; CYP2C19 int: CYP2C19 inhibitor; DZN: DZP: diazepam; FLU: flumazenil; HBD: hydrogen bond donor (optimum: ≤ 5); HBA: hydrogen bond acceptor, (optimum: ≤10); Log P*_o/w_* (MLogP) (optimum: ≤ 5); MF: molecular formula; MR: molar refractivity (optimum: ≤ 140); MW: molecular weight (g/mol) (optimum: ≤ 500); TPSA: topological polar surface area.

#### 3.3.3 ADMET Studies

Our ADMET prediction revealed that CTL has the lowest water solubility (−3.465 log mol/L), which could impact its oral bioavailability. High Caco‐2 permeability indicates good absorption potential in the human intestine, and high human intestinal absorption values suggest that CTL and DZP are likely to be well absorbed orally, while FLU has lower absorption. DZP shows the highest VDss (0.365 log L/kg), indicating extensive tissue distribution. In terms of metabolism, DZP and FLU may interact with other drugs metabolized by CYP1A2, while none of the compounds are CYP2D6 substrates. FLU has the highest clearance rates (1.686 mL/min/kg), suggesting rapid elimination and potential effects on dosing frequency. Additionally, FLU tested positive for AMES toxicity, indicating a potential for mutagenicity (Table 5).

**TABLE 5:** The ADMET data of citronellal, diazepam, and flumazenil.

**Compound name**	**Absorption**			**Distribution**		**Metabolism**		**Excretion**		**Toxicity**	
	Water solubility log S (log mol/L)	Caco‐2 permeability x 10^−6^	Human intestinal absorption (%)	VDss (human) (log L/kg)	BBB permeability (log BB)	CYP450 1A2 inhibitor	CYP450 2D6 substrate	Total clearance (mL/min/kg)	Renal OCT2 substrate	Max. tolerated the dose (log mg/kg/day)	AMES toxicity
CTL	−3.465	1.503	95.359	0.192	0.648	No	No	0.476	No	0.59	No
DZP	−4.196	1.554	97.42	0.365	0.331	Yes	No	0.294	Yes	0.066	No
FLU	−3.621	1.32	93.567	0.101	−0.205	Yes	No	0.758	Yes	0.548	Yes

Abbreviations: CTL: citronellal; DZP: diazepam; FLU: flumazenil.

#### 3.3.4 Toxicity Profile

**FIGURE 4:**
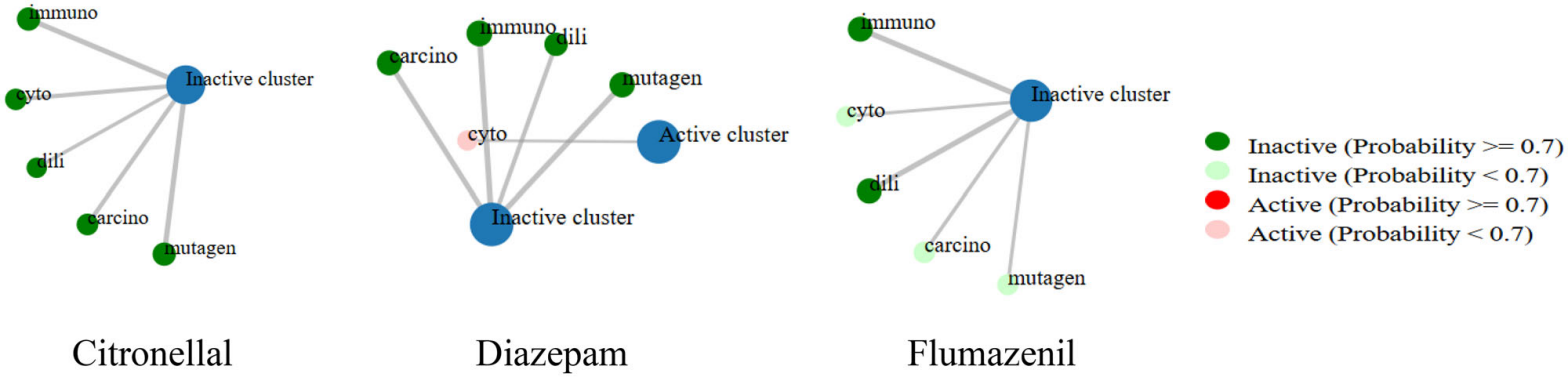
The toxicity network chart of citronellal, diazepam, and flumazenil.

Our computational toxicity analysis classified CTL, DZP, and FLU into toxicity Classes 5, 2, and 4, respectively. DZP showed high toxicity with an LD50 of 48 mg/kg, while CTL and FLU displayed low toxicity. All compounds were inactive for carcinogenicity, immunotoxicity, hepatotoxicity, and mutagenicity. Additionally, DZP was positive for cytotoxicity, whereas CTL and FLU were inactive in this aspect (Table 6 and Figure 4).

**TABLE 6:** Toxicity profile of citronellal, diazepam, and flumazenil with different parameters.

**Ligands**	**Parameters**						
	**Toxicity class**	**LD_50_(mg/kg)**	**Toxicity types**				
			**Carcinogenicity**	**Cytotoxicity**	**Immunotoxicity**	**Hepatotoxicity**	**Mutagenicity**
CTL	5	2420	Inactive	Inactive	Inactive	Inactive	Inactive
DZP	2	48	Inactive	Active	Inactive	Inactive	Inactive
FLU	4	1300	Inactive	Inactive	Inactive	Inactive	Inactive

Abbreviations: CTL: citronellal; DZP: diazepam; FLU: flumazenil.

## 4 Discussion

BDZs are also most commonly used as CNS depressants. These work through the postsynaptic GABA_A_ receptor interaction pathway (Ticku et al. 1983). However, BDZs develop tolerance and physical dependence in patients. For example, DZP produces sedation at 5 to 10 mg in a first‐time user and can develop tolerance among those who repeatedly use it (O'Brien et al. 2006). Therefore, the discovery of new, effective, and safer sedative drugs is a time‐sensitive issue.

Drugs that produce sedative effects can reduce anxiety through calming effects on animals. It is because these can induce normal arousal sleep in animals (Creighton and Lamont 2024). DZP acts through this pathway. In this study, we have also seen that DZP at 2 mg/kg produced a sharp onset of action as well as sleep duration, suggesting its sedative effects in animals. Compared to DZP‐2, CTL at all doses showed low onset of sleep, and its high dose (250 mg/kg) produced slightly higher sleep duration than the DZP‐2 group. Interestingly, with DZP‐2, CTL‐250 significantly (*p* < 0.05) augmented the sleep duration at the highest level among all test groups, denoting that CTL‐250 may have an increasing capacity for DZP's sedative effect in animals. Therefore, our findings might be in agreement with the earlier report of Jäger et al. (1992).

Sedative antidepressants are frequently used in the treatment of primary insomnia. These kinds of drugs, for example, doxepin, mirtazapine, trazodone, trimipramine, and agomelatine, usually promote sleep action through resynchronization of the circadian rhythm (Wichniak et al. 2012). In mammals, the circadian rhythms (e.g., sleep/wake cycles) are regulated by the central circadian clock located in the suprachiasmatic nucleus (SCN), which consists of thousands of individual neurons of the hypothalamus. They synchronize with each other and produce robust and stable oscillations. It is evident that almost all SCN neurons are GABAergic (Ono et al. 2018). In this study, we have seen that CTL at 250 mg/kg doses produced significant sleep duration in comparison to the control as well as the standard groups.

The TS‐induced sleeping protocol is commonly used to investigate the sedative or hypnotic effects of various test substances related to GABA_A_ receptors. TS binds at GABA_A_ barbiturate sites, causing hyperpolarization of the postsynaptic membrane (de la Peña et al. 2013). The BDZ drug DZP potentiates the sedative effect of TS through its agonistic effect on GABA_A_ receptors (Raihan et al. 2011), which causes an influx of chlorine ion (Cl^−^) into the cell (Mula 2016). Therefore, substances that decrease the latency and increase the sleep duration in TS‐induced animals may act on GABA_A_ receptors (Raihan et al. 2011). Thus, the test samples reducing latency and augmenting sleep duration dose‐dependently is a sign of their sedative effects in animals.

Molecular docking predicts ligand–receptor interactions, aiding drug discovery by assessing BA and therapeutic potential (Li et al. 2022). The presence of the same amino acid residues in multiple ligands underscores their crucial role in stabilizing ligand–receptor interactions and enhancing BA (Wootten et al. 2016). CTL interacts with key residues PHE A: 289 and LEU A: 285 on the GABA_A_ receptor, similar to DZP, which has the strongest binding affinity (BA; ‒8.7 kcal/mol). Although CTL shows a weaker affinity (‒5.6 kcal/mol) with no hydrogen bonds, its interactions with critical residues highlight its potential as a candidate ligand for further optimization.

**FIGURE 5:**
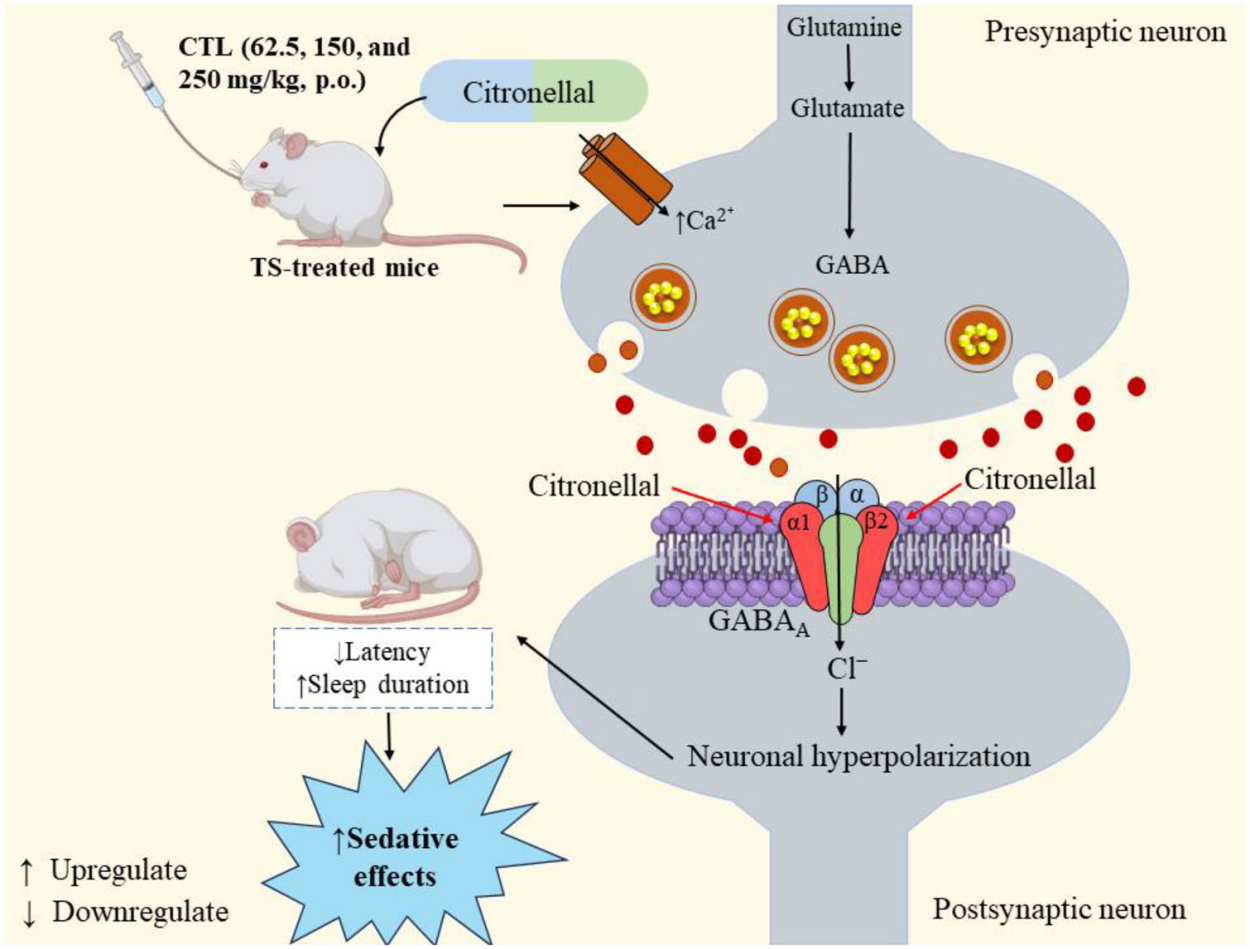
The possible mechanism of citronellal in sedative activity. (The mechanism involves CTL enhancing GABA release by increasing presynaptic calcium influx, leading to GABAergic activation. CTL binds to GABA_A_ receptor subunits [α1 and β2], promoting chloride ion influx, causing neuronal hyperpolarization. This results in reduced sleep latency and increased sleep duration, producing sedative effects.)

Drug‐likeness is a crucial criterion for predicting the “drug‐like” characteristics of a chemical molecule in the early stages of the drug development and manufacturing process. It is assessed by the drug's physicochemical properties, indicating the nature of the drug in relation to pharmacokinetics (Bhuia et al. 2023). One commonly used methodology for assessing pharmacokinetics PKs and drug‐likeness is Lipinski's rule of five, which stipulates that a drug candidate should have a MW of 500 g/mol or less, no more than five HBD, no more than 10 HBA, and a lipophilicity (LogP*_o/w_*) of five or less (Lipinski 2004). In this study, we found that CTL performed as a medicine and showed improved PK characteristics. CTL exhibits high GIA and water solubility, suggesting that it may be well absorbed when taken orally. However, a possible mechanism of CTL in sedative effect is shown in Figure 5.

A critical stage in the creation of new drugs is toxicological screening, which helps identify and prioritize compounds with the highest potential for safe and efficient use in human beings. Additionally, it lessens the possibility of expensive late‐stage losses and improves the curative qualities of already‐existing molecules (Tran et al. 2023). Long‐term chemical exposure typically causes a variety of organ harm in humans, including neurotoxicity, genotoxicity, immunotoxicity, carcinogenicity, and toxicity to the developing and reproductive systems (Sabarwal et al. 2018). In the current study, we predict the drug‐like properties and ADMET features of CTL using the websites SwissADME, pkCSM, and ProTox‐3.0. All estimated parameters for CTL (drug‐like properties and ADMET profile) remained within allowable ranges as Tables 4, 5, and 6 demonstrate. Additionally, CTL has fewer toxicological features and better ADMET profiles.

While this study establishes the sedative potential and acute safety of CTL, several limitations remain. The absence of chronic exposure and repeated‐dose toxicity studies prevents conclusions on long‐term safety and cumulative effects, while the lack of motor coordination and cognitive assessments leaves its impact on these functions unknown. Additionally, the study does not examine sleep architecture, which could be explored through electroencephalography (EEG) studies to assess CTL's effects on rapid eye movement (REM) and non‐REM sleep. The absence of PK data, including bioavailability and metabolism, also limits its therapeutic relevance. Furthermore, potential tolerance and dependence with prolonged use remain unexplored, necessitating future studies to determine whether CTL maintains its efficacy over time without withdrawal effects. Although this study provides indirect evidence of CTL's interaction with the GABAergic system through its combination with DZP and FLU, the lack of direct experimental validation remains a limitation. Without electrophysiological studies, such as patch‐clamp recordings, or receptor binding assays, it is not possible to confirm whether CTL directly modulates GABA_A_ receptor activity or acts through an indirect mechanism. This limitation highlights the need for future studies to assess CTL's functional effects on GABA_A_ receptor currents and BA, which would provide stronger mechanistic support. Understanding the precise mode of action is crucial for developing CTL as a potential sedative agent and differentiating it from existing GABAergic drugs in terms of efficacy, specificity, and safety.

## 5 Conclusion

CTL exhibited dose‐dependent sedative effects in *Swiss mice*, significantly reducing sleep onset (*p* < 0.05) and increasing sleep duration at 250 mg/kg, compared to controls. It enhanced sleep when combined with the GABAergic agonist DZP and reversed the effects of the antagonist FLU, confirming its GABAergic mechanism. In vitro, CTL and DZP significantly increased GABAergic activity, with CTL showing 71.43% activity at 250 µg/mL, while FLU reduced GABAergic activity at higher concentrations. In silico studies revealed strong BAs of CTL for GABA_A_ receptor subunits α1 and β2 (‒5.6 kcal/mol). Acute toxicity tests showed no adverse effects up to 2000 mg/kg, supporting the safety of test doses up to 250 mg/kg. Collectively, these findings suggest that CTL exerts sedative effects by modulating GABA_A_ receptors, warranting further studies to confirm its molecular mechanisms.

## Author Contributions

**Md. Torequl Islam**: conceptualization, investigation, methodology, data curation, visualization; writing–original draft, writing–review and editing, formal analysis. **Md. Sakib Al Hasan**: data curation, investigation; writing–original draft, software. **Emon Mia**: data curation, software. **Irfan Aamer Ansari**: software, writing–original draft. **Siddique Akber Ansari**: software, writing–original draft, resources, funding acquisition. **Md. Amirul Islam**: writing–review and editing, validation, visualization. **Md. Saifuzzaman**: writing–review and editing, project administration, supervision, validation.

## Ethics Statement

This study was approved by the Animal Ethics Committee of Khulna University (KUAEC‐2023‐05‐09). No anesthetic or surgical procedures were used for this study. We checked only the behavioral parameters after the treatment; therefore, this study did not require any medication to overcome or reduce the suffering of the experimental animals.

## Conflicts of Interest

The authors declare no conflicts of interest.

### Peer Review

The peer review history for this article is available at https://publons.com/publon/10.1002/brb3.70446.

## Data Availability

Data will be made available on request.
